# Motor imagery for paediatric neurorehabilitation: how much do we know? Perspectives from a systematic review

**DOI:** 10.3389/fnhum.2024.1245707

**Published:** 2024-03-20

**Authors:** Amalia Egle Gentile, Sergio Rinella, Eleonora Desogus, Cristiano Maria Verrelli, Marco Iosa, Vincenzo Perciavalle, Martino Ruggieri, Agata Polizzi

**Affiliations:** ^1^National Centre for Rare Diseases, Istituto Superiore di Sanità (ISS), Rome, Italy; ^2^Department of Educational Science, Chair of Pediatrics, University of Catania, Catania, Italy; ^3^Department of Electronic Engineering, University of Rome Tor Vergata, Rome, Italy; ^4^Department of Psychology, Faculty of Medicine and Psychology, Sapienza University of Rome, Rome, Italy; ^5^Santa Lucia Foundation (IRCCS), Rome, Italy; ^6^Faculty of Medicine and Surgery, Kore University of Enna, Enna, Italy; ^7^Unit of Clinical Pediatrics, Department of Clinical and Experimental Medicine, University of Catania, Catania, Italy

**Keywords:** motor imagery, neurorehabilitation, neurodevelopment, developmental coordination disorder (DCD), cerebral palsy, autism spectrum disorder (ASD), intellectual disabilities (ID)

## Abstract

**Background:**

Motor Imagery (MI) is a cognitive process consisting in mental simulation of body movements without executing physical actions: its clinical use has been investigated prevalently in adults with neurological disorders.

**Objectives:**

Review of the best-available evidence on the use and efficacy of MI interventions for neurorehabilitation purposes in common and rare childhood neurological disorders.

**Methods:**

systematic literature search conducted according to PRISMA by using the Scopus, PsycArticles, Cinahl, PUBMED, Web of Science (Clarivate), EMBASE, PsychINFO, and COCHRANE databases, with levels of evidence scored by OCEBM and PEDro Scales.

**Results:**

Twenty-two original studies were retrieved and included for the analysis; MI was the unique or complementary rehabilitative treatment in 476 individuals (aged 5 to 18 years) with 10 different neurological conditions including, cerebral palsies, stroke, coordination disorders, intellectual disabilities, brain and/or spinal cord injuries, autism, pain syndromes, and hyperactivity. The sample size ranged from single case reports to cohorts and control groups. Treatment lasted 2 days to 6 months with 1 to 24 sessions. MI tasks were conventional, graded or ad-hoc. MI measurement tools included movement assessment batteries, mental chronometry tests, scales, and questionnaires, EEG, and EMG. Overall, the use of MI was stated as effective in 19/22, and uncertain in the remnant studies.

**Conclusion:**

MI could be a reliable supportive/add-on (home-based) rehabilitative tool for pediatric neurorehabilitation; its clinical use, in children, is highly dependent on the complexity of MI mechanisms, which are related to the underlying neurodevelopmental disorder.

## Introduction

*Motor imagery* (MI) is an active cognitive process (a dynamic state), during which an individual internally rehearses or simulates (within its working memory) a given action, without *movement execution* (ME) (Decety and Grèzes, 1999; Jeannerod, 2001; Yap and Lim, 2019). Defined as a mental representation of an action without its actual performance, MI straightforwardly includes the “image” of planning, modulation and execution of movements (Decety, 1996).

MI is usually named *explicit* when the representation of the task is consciously reproduced by individuals, while it becomes *implicit* when the task is unconsciously reproduced.

In any case, this intricate process, occurring within the motor domain, is conceptually corresponding to an internal model of motor/behavioral representation, allowing the possibility that a cognitive simulation of an action sorts out an effective activation of the motor pathways as the real movement does. This individual ability led to the use of MI to improve motor performance and to learn or re-learn motor skills for neurorehabilitive purpose (Simonsmeier et al., 2021).

Over time, MI, taken as a complex cognitive process, has turned up to be an intriguing field of research as well as a practical tool in sportive training, not only for the beneficial consequences on performance outcomes, but also for its positive results on psychological outcomes including affective and motivational effects (Guillot and Collet, 2008; Cumming and Ramsey, 2009; Frank et al., 2016; Simonsmeier et al., 2021).

As it occurs with motor tasks, MI represents the physiological result of complex sensory-motor integrations of feedforward and feedback to and from the external environment. In fact, during movements sensory-motor pathways connect the primary motor cortex, the premotor cortex and the supplementary motor area to the parietal somatosensory cortex, the cingulate cortex, the striatal pathway and the cerebellum. Whilst the former circuits are involved in generating movements, preparing muscles and stabilizing posture and coordination, the latter (afferent circuits) control execution and functions such as motor planning, motivation, decision and work memory. Thus, MI relies on complex and bidirectional sensorimotor information to create a mental simulation of a task (Yip and Lui, 2023).

*Action observation* (AO) is, instead, a cognitive perceptual process, during which an individual observes a purposeful action, performed by others. Experimental and clinical interest in AO rose, following the identification of the *mirror-neuron system* (MNS). As it occurs with simulation of actions, MI and AO involve the kinesthetic, visual and spatial aspects of the corresponding action.

Various studies revealed that MI, AO and motor execution (ME) approximately share the same neural networks activated during motor performance (Crammond, 1997; Grèzes and Decety, 2001; Jeannerod, 2001; Hardwick et al., 2018) as demonstrated by evidences coming from preclinical researches in neurophysiology (Fleming et al., 2010; Hardwick et al., 2018; Kurkin et al., 2023) together with results of human experiments carried out with transcranial magnetic stimulation and functional MRI.

Few but relevant age-related studies disclosed that the ability of MI matches, from some point of view, on the trajectory of motor and sensory development so that compounds of the internal action control model are acquired during development through motor learning and sustained by experiences which provide sensory, kinesthetic and visuo-spatial feed-back. In normally developing children, the emergence of MI skills seems to occur explicitly at around age 5 to 6 years and then develops with age, being refined between early adolescence and early adulthood (Gabbard and Bobbio, 2011; Souto et al., 2020b; Saleem, 2023).

More extensive explanations of MI in children direct attention—as in adulthood—to a multidimensional construct of the phenomenon where complex sensory-motor data are efficiently integrated to mentally set out an action. Maturation of the parietal and frontal cortices structure and function contribute, alongside with practice, to the spontaneous progression of motor abilities control during development (Skoura et al., 2009). A background of theoretical models supports this assumption as nicely reviewed by Saleem (2023).

Beside the evidence that imagery can promote motor learning in young athletes (Simonsmeier et al., 2018), more in general, MI training has been successfully showed to positively influence motor learning in healthy children and adolescents (Behrendt et al., 2021).

Upon these bases, it was obvious that imagery training could appropriately contribute, even more so, in enhancing or resuming the motor network in some pathological conditions of the nervous system of pediatric onset (Rannaud Monany et al., 2022).

Studies on MI in children with development coordination disorders (DCD), a complex paradigm of atypical neurodevelopment, disclosed a reduced capacity to use the internal modeling of motor representation to reach aspect of motor control, planning and execution (Steenbergen et al., 2020). Other instances of congenital abnormalities of neurodevelopment, are likely accompanied by a dysfunction of the internal motor representation process leading to an altered MI, AO and ME (Mutsaarts et al., 2006; Crajé et al., 2010; van Elk et al., 2010; Steenbergen et al., 2013; Jongsma et al., 2016; Errante et al., 2019).

A deficit of the internal modeling of motor representation and defective MI tasks is then conceivable in those neurological conditions of infantile onset where the motor domain including the movements execution (ME) are compromised early, since prenatal age, no matter what the severity of motor dysfunction is.

Apparently, explicit MI ability could be instead preserved in some cases of cerebral palsy, arguing that the capability to retrieve motor representations is maintained in some children with compromised motor abilities of various degree (Errante et al., 2019). Perhaps, this can happen when the cause of the disorder does not hinder the motor trajectory in the very early stages of development, but during late prenatal or postnatal life.

Accordingly, studies conducted in adulthood patients who underwent MI training after post-stroke brain injuries, disclosed that MI tasks is influenced by the internal representation of the motor act, regardless the level of the individual residual motor function (Sharma et al., 2006; Schulz et al., 2019).

At large, it arises that MI training can contribute, at any age, to the amelioration of motor activity in term of motor learning in health status or habilitation/rehabilitation of neurological dysfunctions, as the internal models of motor representation support MI, AO as well as motor execution (ME) (Rannaud Monany et al., 2022).

The possibility to evaluate the quality of MI is therefore useful to examine motor representations and to predict the leeway for feasible therapeutic interventions. For its implications, imagery-based techniques are so far limitedly used in rehabilitation to stimulate neuromuscular pathways and gain access to the motor network of persons with neurological disorders. Over the last years research has advanced the therapeutic potential of MI interventions in adults (vs. children) with neurological disorders. Indeed, for adults’ conditions such as stroke, motor coordination disorders, Parkinson’s syndromes, dystonia, multiple sclerosis, brain and spine trauma, complex regional pain, etc., (Morya et al., 2019), various approaches using MI are reasonably employed as adjunctive tools to conventional neurorehabilitation or in combination with newer interventions (i.e., music therapy) (Adams et al., 2014; Haire et al., 2021).

The present review aims to systematically analyze MI based clinical studies in childhood and adolescence with various dysfunction of the nervous system to investigate the efficacy of using MI in the pediatric (neuro)rehabilitative practice.

## Methods

**Figure 1:**
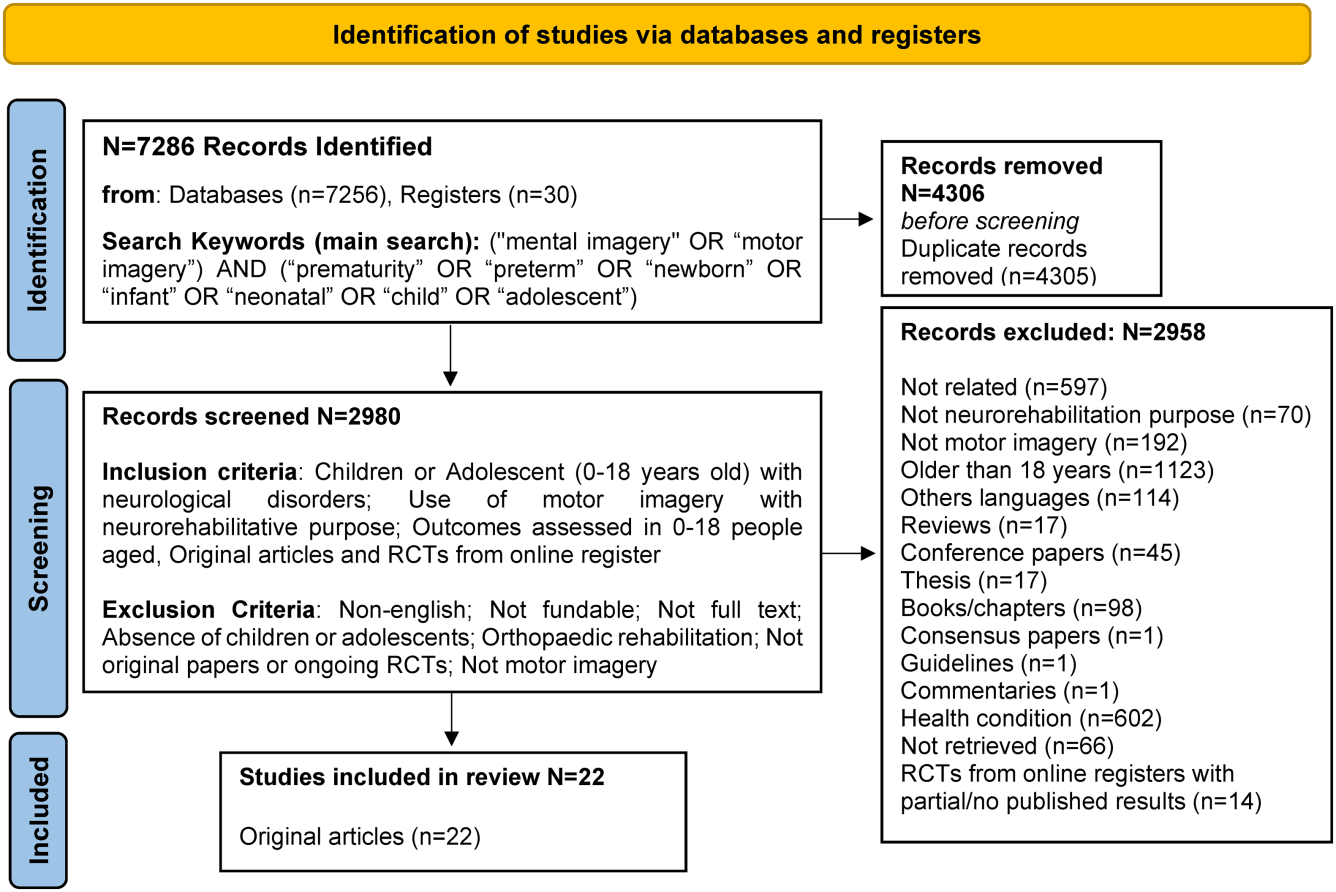
Review flow diagram according to the PRISMA statement 2020.

The systematic review was conducted according to the PRISMA statement (see Figure 1). Studies published up to December 30, 2022, were retrieved through a literature search in the Scopus, PsycArticles, Cinahl, PUBMED, Web of Science (Clarivate), EMBASE, PsycInfo, and COCHRANE databases. Where available, “0–18 years “age filters were used and applied for PubMed and MeSH terms. The search terms were: (“mental imagery” OR “motor imagery”) AND (“prematurity” OR “preterm” OR “newborn” OR “infant” OR “neonatal” OR “child” OR “adolescent”). Duplicates were removed.

Articles meeting the following criteria were included: (1) reference population of children or adolescents (0–18 years) with neurological disorders; (2) use of MI for neurorehabilitation purposes; (3) outcomes assessed in the age range between 0–18 years; (4) original articles; (5) English language; (6) full text articles available. Three independent reviewers screened full-text articles and extracted the identified data using a standardized collection form. Disagreements on article inclusion were resolved by consensus obtained by two additional authors. All researchers confirmed the final evaluation. The level of evidence was assigned for each retrieved work, in accordance with the Howick et al. (n.d.). All retrieved RCT studies were scored by using the PEDro Scale (Cashin and McAuley, 2019).

Given the heterogeneity of the studies reviewed, a qualitative assessment of the results was chosen. In Table 1, a binary variable (Y/N) has been assigned based on explicit statements of efficacy by the respective authors. References were handled with Zotero (ver. 6.0.18).

**Table 1:** Summary table of characteristics of the included studies.

Neurodevelopmental conditions	References	Study design	Level of evidence	PEDro SCORE	Participants			MI intervention			Measurement tools (pre- and/or post-intervention)	Outcome		Efficacy
					Sample size/No. (sex)	Age range (y.o.)	Control group	Treatment duration/sessions	Tasks	Type		Evaluation time	Assessment^b^	
ADHD	Chevalier et al. (2003)	Concurrent cohort study	3	/	12 (n/a)	6–9	Y	6 months n/a sessions	Visual-motor imagery exercises	Ad hoc	DuPaul Diagnostic Questionnaire; Attention Education Program (AEP); Conners Continuous Performance Test (CPT)	Pre-post at follow-up after 6 months	Teachers focus groups (see *Measurements column*)	Y
ASD	Xie et al. (2022) ^a^	Quasi-experimental	3	/	20 (ASD group: 16M, 4F) 20 (ID group: 16M, 4F)	7–15	Y	1 session	Mental simulation of on-demand actions	Ad hoc	The Raven Standard Progressive Matrices Test (SPM); Peabody Picture Vocabulary Test Revised, Chinese Version (PPVT-R); Action sequences span paradigm scores	Pre-post	Action sequences span paradigm scores	N
CP	Taherian et al. (2017)	Before-and-after study	4	/	6 (3M, 3F)	7–43	N	3 weeks 5–7 sessions	Moving virtual object (MI-BCI: Emotiv EPOC)	Ad hoc (Digital)	EEG; Game scoring	Pre-post	(See *Measurements column*)	N
	Cabral-Sequeira et al. (2016)	Quasi-experimental	3	/	31 (16 M, 15F)	11–16	Y	2 days 2 sessions/day	Modelling (mental simulation of observed action)	*Ad hoc*	Kinematic analysis	Pre-post	(See *Measurements column*)	Y
	Souto et al. (2020a,b)	Controlled before-and-after study	3	/	24 (13M, 11F)	7–14	Y	8 weeks 16 sessions	Modelling (mental simulation of observed action)	Ad hoc	Raven’s Coloured Progressive Matrices test; Block Design subtest of the Wechsler Intelligence Scale; backward Digit Span and backward Corsi Cubes tests; Manual Ability Classification System (MACS); Assisting Hand Assessment (AHA) version 4.3	Pre-post At follow-up after 8 weeks	Assisting Hand Assessment (AHA) version 4.3	Y
	Xie et al. (2021)	Before-and-after study	4	/	10 (5M, 5F)—short term group 8 (7M, 1F)—long term group	8–12	/	12 weeks (short term) 60 weeks (long term) n/a sessions	Mental simulation of on-demand actions (MI-BCI)	Ad hoc	EEG	Pre-post	(See *Measurements column*)	Y
	Stefano Filho et al. (2021)	Before-and-after study	4	/	5 (n/a)	11–14	/	n/a weeks 1 session	First-person avatar walking (VR-based Motor imagery: Oculus rift)	Ad hoc (Digital)	EEG	Pre-post at follow-up after 1 year	(See *Measurements column*)	/
	Gözaçan Karabulut et al. (2022) (only abstract available)	RCT	2	n/a	n/a	n/a	Y (two control groups: CP + TD)	8 weeks n/a sessions	MI + CT (Motor Imagery training: n/a)	n/a	Movement Imagery Questionnaire-For Children (MIQ-C), mental chronometry, functional mobility, and resting muscle activation	n/a	(See *Measurements column*)	Y
CRPS	Hayashi et al. (2016)	Case-report	4	/	1F	15	/	80 days n/a sessions	Mirror action mental simulation	Ad hoc	Visual analog scale (VAS); Neglect-like symptoms (NLS) questionnaire; Pain Catastrophizing Scale (PCS); Barthel Index	Pre-post	(See *Measurements column*)	Y
	Tubic (2018)	Case-report	4	/	1F	11	/	12 weeks n/a sessions	Graded Motor Imagery (GMI: App Recognize Back)	Conventional (Digital)	Clinical evaluation	Pre-post at follow-up after 3.5 months	(See Measurements column)	Y
DCD	Wilson et al. (2016)	RCT	2	9/11	12 (n/a) + 12 (n/a)	7–12	Y	5 weeks 1 session/week	Visual imagery and modelling	Ad hoc	Background parents’ questionnaire on history of neurological conditions; Movement Assessment Battery for Children (MABC)	Pre-post	MABC	Y
	Adams et al. (2017)	Quasi-experimental	3	/	4 (n/a)	7–12	Y	9 weeks 1 session/week	Visual imagery and modelling	Ad hoc	Movement Assessment Battery for Children (m-ABC-2); Motor Coordination Questionnaire (MCQ)	Pre-post	Therapists’ Experiences (see *Measurements column*)	Y
	Scott et al. (2019)	Case-control study	4	/	12 (11M, 1F)	7–12	Y	n/a	AO + MI (mental simulation of observed daily action)	Ad hoc	Movement Assessment Battery for Children (m-ABC-2); Movement imagery questionnaire-3; Developmental Coordination Disorder Questionnaire 2007; Vanderbilt Attention-Deficit Hyperactivity Disorder (ADHD) Diagnostic Parent Rating Scale	n/a	(See *Measurements column*)	Y
	Marshall et al. (2020)	RCT	2	6/11	20 (13M, 7F)	7–11	/	n/a weeks 1 session	AO + MI (virtual radial Fitts task: Unity3D)	Conventional (Digital)	eye-tracking; software recording	Pre-post	(See *Measurements column*)	Y
	EbrahimiSani et al. (2020)	Controlled before-and-after study	3	/	40F	7–10	Y	8 weeks n/a sessions	VR-based Intervention	Ad hoc (digital)	Time recording; hand rotation task, anticipatory action planning task, Rapid online control task; software recording	Pre-post	(See *Measurements column*)	Y
	Scott et al. (2020)	Case-control study	4	/	13 (6M, 7F)	7–11	Y	1 session	AO + MI (mental simulation of observed daily action)	Ad hoc	m-ABC-2; Movement imagery questionnaire-3; health questionnaire and Vanderbilt ADHD Diagnostic Parent Rating Scale	n/a	(See *Measurements column*)	Y
ID	Hemayattalab and Movahedi (2010)	RCT	2	8/11	40 (n/a)	12–15	Y	n/a weeks 24 sessions	Sport-related mental simulation (mental simulation of an action)	Ad hoc	EMG	Pre-post at follow-up after 10 days	Tuki follow up test (see *Measurements column*)	Y
	Chen et al. (2015)	RCT	2	7/11	91 (50M, 41F)	6–12	Y	16 weeks 3 sessions/week	Modelling (mental simulation of an observed action)	Ad hoc	Study-specific questionnaire included child’s anthropometric variables, demographic data, received medications, treatments, and paramedical therapies; Test of Visual Perceptual Skill-third edition (TVPS-3); Wisconsin Card Sorting Test 64-card version (WCST-64); Stroop Color–Word Test, children’s version; Caregivers’ diaries for describing learning effects	Pre-post	(See *Measurements column*)	Y
	Xie et al. (2022) ^a^	Quasi-experimental	3	/	20 (ASD group: 16M, 4F) 20 (ID group: 16M, 4F)	7–15	Y	1 session	Mental simulation of on-demand actions	Ad hoc	The Raven Standard Progressive Matrices Test (SPM); Peabody Picture Vocabulary Test Revised, Chinese Version (PPVT-R); Action sequences span paradigm scores	Pre-post	Action sequences span paradigm scores	N
NP	Casanova-García et al. (2015)	RCT (preliminary study)	2	4/11	40 (all)	5–18	Y	4 weeks Motor imagery exercises (5 days/week for 1 week) + Laterality recognition (5 days/week for 1 week) + Mirror therapy (5 days/week for 2 weeks)	Graded Motor Imagery (GMI)	Conventional	Visual analog scale (VAS); survey on catastrophizing; accelerometry for physical activity	Pre-post	(See *Measurements column*)	Y
SRBC	Gagnon et al. (2016)	Before-and-after study	4	/	10 (7M, 3F)	14–18	/	6 weeks	Mental simulation of an action	Ad hoc	Pictorial Children’s Effort rating Table (P-CERT); Post-Concussion Scale; Beck Depression Inventory-Second Edition; Pediatric Quality of Life; Multidimensional Fatigue Scale; Bruininks-Oseretsky Test of Motor Proficiency – Second Edition (BOT); cognitive functioning (ImPACT®); State Trait Anxiety Inventory (STAI)	Pre-post	(See *Measurements column*, except for the *P-CERT*)	Y
ST	Lu et al. (2020)	Before-and-after study	4	/	26 (n/a)	16–70	/	6 weeks n/a sessions	Mental simulation of affected limb action (MI-BCI; EEG system)	Ad hoc (Digital)	EEG; ROM; Mini-Mental State Examination (MMSE); Barthel index	Pre-post	(See *Measurements column*)	Y
SY	Biglioli et al. (2017)	Cases series	4	/	18 (12M, 6F)	12–47	/	3 weeks n/a sessions	Mental simulation of on-demand actions	Ad hoc	EMG; HouseeBrackmann (HB) scale	Pre-post At follow-up after 3 weeks	(see *Measurements column*)	Y

Neurodevelopmental conditions (in alphabetical order); ADHD, attention deficit hyperactivity disorder; ASD, autism spectrum disorders; CP, cerebral palsy; CRPS, complex regional pain syndrome; CT, conventional therapy; DCD, developmental coordination disorder; ID, intellectual disabilities; NP, neuropathic pain (due to cancer); SRBC, sports-related brain concussion; ST, stroke; SY, synkinesis; TD, typical development; n/a, not available (not described data in the article).

a The article refers to both ASD and ID conditions.

b In cases of overlap, please refer to the “Measurement Tool” column.

As indicated in Figure 1, clinical trials (CTs) retrieved from online registries (*clinicaltrials.gov*, *trialsearch.who.int*, and *clinicaltrialsregister.eu*) were excluded from the analysis as they refer to either partial published results (on adulthood only), or to still ongoing or unknown results from studies on pediatric population. However, for their potential relevance in deeping knowledge in a research and clinical field with a general lack of robust evidence, available data from these CTs consisting in 11 randomized CTs (RCTs) and 3 non-RCT studies are included as Supplementary material (S1) and at times resumed throughout the discussion.

## Results

In total, 22 original research clinical studies met the defined inclusion criteria (Figure 1). The main characteristics of these 22 original studies are shown in Table 1 and describe below.

A population of 476 individuals was collected from all records with the exclusion of 1 study where the sample size was not available (Gözaçan Karabulut et al., 2022).

MI was investigated and applied as a unique or complementary rehabilitative treatment in individuals with 10 different congenital or acquired neurological conditions. Intellectual disabilities (ID) (*N* = 151), development coordination disorders (DCD) (*N* = 113) and cerebral palsies (CP) (*N* = 84) were the most frequently studied conditions, followed by neuropathic pain (NP) (*N* = 40), stroke (ST) (*N* = 26), autism spectrum disorders (ASD) (*N* = 20), synkinesis (SY) (*N* = 18), attention deficit hyperactive disorder (ADHD) (*N* = 12), sports-related brain concussion (SRBC) (*N* = 10), and complex regional pain syndrome (CRPS) (*N* = 2).

The overall sample size of the intervention groups was highly variable ranging from single case reports to a cohort of 91 patients. In almost all studies a control group was included. Individuals were aged from 5 to 18 years. Three studies endeavored also adult populations (Biglioli et al., 2017; Taherian et al., 2017; Lu et al., 2020). Participants were mostly males whilst in 8 records sex was unknown or not specified.

Apparently, the overall length of intervention with MI overlapped with the total length of intervention of MI coupled to common rehabilitative program. Treatment was extended from 2 days to 6 months, duration was unknown in 3 studies (Hemayattalab and Movahedi, 2010; Scott et al., 2019, 2020). Rehabilitative exercises based on MI tasks were run from 1 to 24 sessions. MI tasks were distinguished between conventional and more frequently purposeful ad-hoc tasks. Conventional tasks, mostly driven by the principles of Graded Motor Imagery (GMI) programs, were detailed only in a few cases and consisted *mainly* in graded MI interventions, Radial Fitts Task and Hand Rotation Task. Ad-hoc tasks essentially laid into MI exercises of on-demand actions, modelling (mental simulation of AO without clear relation to the active observation) and MI + AO (AO tasks are explicitly spelled out). Digital tools were employed in 6 studies, under the umbrella of brain computer interfaces, virtual reality, App Recognize Back^™^ (Taherian et al., 2017; Tubic, 2018; EbrahimiSani et al., 2020; Lu et al., 2020; Marshall et al., 2020; Stefano Filho et al., 2021). In a few studies, MI tasks were not indicated. Measurements of MI were obtained through different tools including movement assessment batteries, mental chronometry tests, scales and questionnaires for MI and for neurodevelopmental disorders, EEG and EMG. Clinical outcome was assessed at varying intervals of follow-up. Evaluation of changes in physical/motor and cognitive parameters was reported in 21/22 original articles, with the exception of one study for which only the abstract was available (Gözaçan Karabulut et al., 2022). Considering that the effectiveness of MI differs for each developmental disorder/condition, the use of MI was stated effective in 19/22, while in the remnant studies efficacy was uncertain (Table 1).

## Discussion

This systematic review focuses on how MI, through precise training, can drive gaining or re-gaining of abilities in children with various neurodevelopmental conditions. The analysis, which counted 22 original research studies for a total of 476 participants demonstrates a definite interest in research and practice in exploring the efficacy of MI as neurorehabilitative intervention in the pediatric age.

In this respect, MI could represent an opportunity not only for studying the mechanisms that may underlie MI responses but also for boosting treatment outcomes in children with neurodevelopment conditions.

We found that treatments based on MI interventions have been addressed to a mixed group of congenital and acquired disorders of the nervous system (e.g., DCDs, stroke, CP, ID, ASD, ADHD). Participants were rather equally divided in control groups (a small number of studies included only the experimental group), which only received traditional physical therapy and experimental groups, that received MI interventions, only in a few cases there was a combination of both (see Table 1).

Due to the multidimensional and multimodal construct of imagery and its dual perspective (internal and external), assorted and integrated strategies were developed to engage different aspects of the imagery ability.

So, patients’ performance was measured through various assessment pre- and post-intervention, and at a follow-up session. The great majority of the reviewed experimental studies examine the short-term effects of MI in children aged 5–18 years. In one instance only, focusing on CP, effects were evaluated at a year follow-up (Table 1).

In details, 6 original articles investigated MI interventions in children with DCDs (Wilson et al., 2016; Adams et al., 2017; Scott et al., 2019; EbrahimiSani et al., 2020; Marshall et al., 2020; Scott et al., 2020).

Although poor motor planning is a key feature of children with DCDs (Bhoyroo et al., 2019), in most of the works, ad-hoc MI techniques stimulating motor and sensory modalities were used. Interventions include both visual imagery and modeling (Wilson et al., 2016 Adams et al., 2017), MI plus AO (Scott et al., 2019, 2020; Marshall et al., 2020) or VR-based training through the “Kinect games” of the Xbox 360 (EbrahimiSani et al., 2020). Visual imagery and modeling allowed to associate motor imagery training (MIT) to perceptual-motor training (PMT) to enhance the acquisition of motor skills (Wilson et al., 2016) or adding the occupational therapy, to improve imitation skills (Adams et al., 2017). In both studies, MIT was as effective as conventional physical therapies, resulting in significant improvements in motor scale scores. In one additional study, the benefits of VR-based training on predictive motor control, through the “Kinect games” of the Xbox 360 have been evaluated (EbrahimiSani et al., 2020). Results showed that MIT significantly improved both motor planning and predictive motor control skills using the VR-based training allowing also the maintenance of its effectiveness even at 2 months follow-up. Three further studies explored the effectiveness MIT (Mental Motor Simulation; Virtual radial Fitts task) combined to the Action Observation (AO): some benefit was recorded in reducing the deficits of internal modeling and eye-manual coordination (Marshall et al., 2020) and in enhancing automatic and intentional imitation in children with DCDs (Scott et al., 2019, 2020). The results of the three studies showed that the combination of AO + MI was more effective than AO alone in improving intentional and automatic imitative abilities and in enhancing response times, eye-to-manual coordination and fluidity of motion kinematics. The use of MI combined with AO shows the greatest evidence of treatment effectiveness. At the same time, the use of VR is considered a valuable aid to set up MIT programs.

Children with CP get into troubles with MI compared to peers with typical development, even if this is not totally compromised (Steenbergen et al., 2007; Souto et al., 2020b; Williams et al., 2021). Other evidences have focused instead on assessing the value of both implicit and explicit MI functions (Parsons et al., 1995; Parsons, 2001). In this respect, some studies (Spruijt et al., 2013; Molina et al., 2015; Lust et al., 2016; Errante et al., 2019) concluded that children and adolescents with CP show deficits in tasks requiring the use of implicit MI, while explicit MI ability appears to be preserved. This suggests that working on explicit MI can be useful for rehabilitating motor function. More recently Williams et al. (2021) nicely showed that MI deficits are not universally observable in children with congenital hemiplegia and that, for example, the performance of the hand laterality task (HLT) can be as fast and accurate as typically developing peers. The poor performance on the HLT was rather irrespective of the affected side as previously stated (Mutsaarts et al., 2007), depending more on the level of daily functioning of the hand. Moreover, the impact of low IQ on test performance should be believed a bias (Williams et al., 2021). These considerations raise the importance of evaluating individual performance and characteristics before drawing general conclusions on the estimation of the efficacy of MI training in children with CP.

The revised 6 original papers on CP revealed the prevailing use of MIT often in combination with AO (Cabral-Sequeira et al., 2016; Taherian et al., 2017; Souto et al., 2020a; Xie et al., 2021; Gözaçan Karabulut et al., 2022). Stefano Filho et al. (2021) proposed in their study a combined AO and MI VR intervention proving that detectable changes in functional connectivity (FC) patterns are partially due to the AO + MI VR task that the patients performed.

All the interventions proposed in these studies showed to be effective, with the exception of the study by Taherian et al. (2017), who obtained inconclusive results due to the instability in using the device, with the authors hoping for greater material flexibility for future products.

Items investigated were gait and capacity of the lower limbs, specific kinematics functions, balance and trunk resistance. Besides MI tasks, the BeFAST method (Brain Change After Fun, Athletic, Sports-skill Training), the PETTLEP model which takes into account many different domains related to motor imagery: physical features, environment, task-related aspects, timing equivalence, learning, emotion, and perspective (Morone et al., 2022) together with MI exercises were applied.

Three original papers (Xie et al., 2022) including 2 RCTs (Pedro score of 8/11 and 7/11 respectively) (Hemayattalab and Movahedi, 2010 Chen et al., 2015) considered ad-hoc MI techniques based on modeling to support children and adolescent with ID. The age of the participants ranged from 6 to 15 years. Overall treatment duration ranged from 1 single session to 3 sessions1/week for 16 weeks.

The works used ad-hoc MI techniques for the treatment, which included exercises of mental simulation of observed or requested actions. Assessments of the patients’ MI skills were not foreseen, while anthropometric and physiological parameters, scales to assess motor skills and cognitive functioning were used to evaluate the efficacy of the treatment. Instrumental tools included the Test of Visual Perceptual Skill-third edition (TVPS-3), the Wisconsin Card Sorting Test 64-card version (WCST-64), the Stroop Color-Word Test, children’s version, and caregiver diaries with notes on observed learning progress. Only one study had positively evaluated MI intervention Chen et al. (2015) and Xie et al. (2022) found that only typically developing children performed better following MI intervention, while Hemayattalab and Movahedi (2010) found that MI alone was effective when compared to the group without any treatment, but less effective than those who carried out physical practice only.

Despite the considerable attention paid today to many aspects of the ASD, relatively few experimental studies considered MI processes in ASD (Conson et al., 2013; Chen et al., 2018; Piedimonte et al., 2018). The prevalence of motor difficulties in children with ASD is quite high as well as their influence on behavioral and daily functions (Lim et al., 2021). As a matter of fact, an impaired imitation has been discovered to be a relevant factor contributing to social communication deficits (Dowd et al., 2010). Accordingly, the neural correlate of imitation, the mirror neuron system (MNS), is assumed to be dysfunctional in ASD, ensuing deficit of imitation as one of the crucial behavioral features in ASD (Chan and Han, 2020). It is well-known that the MNS is involved in the imitation of movements, but also in action recognition, MI and motor learning process (Johansson et al., 2022). When MI was explored using the HLT task to investigate the development of MI in children with ASD, results showed a performance variability in the affected group with more deficits than the control group in the MI criterion task (Conson et al., 2013). Hence, in children with ASD, there is either a clear failure or a delay to develop motor representation. This inability is significant for the construction of the model of body movements during action and could be responsible for a series of clinical features related to motor disturbances. In this respect, children with ASD may take an advantage from *ad hoc* neurorehabilitative training also to help learning of novel motor actions. On this topic, a quasi-experimental study (Xie et al., 2022) was reviewed. Unfortunately, ad-hoc exercises of mental simulation of actions failed to demonstrate any type of efficacy as stated by Xie et al. (2022).

In their cohort of children with ADHD, Chevalier et al. (2003), tested the effectiveness of the Attention Education Program (AEP), comprising visual MI techniques, measured through the DuPaul Diagnostic Questionnaire and the Conners Continuous Performance Test (CPT). The results showed an improvement in reaction times and a reduction in task errors. The ongoing clinical trial (NCT05208255, 2022) targets the effects of telerehabilitation-based exercise and MI practices on symptoms and balance skills. MI training is performed remotely in the form of imagined Neurocognitive Exercise Program (NEP), a multimodal exercise program including different motor coordination exercises and cognitive tasks, for a total length of 6 weeks (2 sessions/week).

The rational of MI intervention in CRPS is the re-organization of the primary sensory cortical and associated motor areas by means of MIT. Two case reports of CRPS were reviewed disclosing in either case, an improvement in pain levels and motor performances (Hayashi et al., 2016 Tubic, 2018). The ages of the participants in the two studies were 15 and 12 years, respectively. The interventions lasted 80 days (Hayashi et al., 2016) and 12 weeks (Tubic, 2018). Hayashi et al. (2016) proposed an intervention based on *ad-hoc* mirror action mental simulation techniques, the efficacy of which was measured using the Visual analog scale (VAS), Neglect-like symptoms (NLS) questionnaire, Pain Catastrophizing Scale (PCS) and the Barthel Index. Differently, Tubic (2018) experimented a combined intervention of epidural infusions of analgesics and MI techniques based on Graded Motor Imagery (GMI), administered via a mobile App (App Recognize Back). In either case, authors described an improvement in pain levels.

The results of a pilot RCT (Pedro score of 4/11) on NP were reported by Casanova-García et al. (2015). The intervention involved 40 patients aged between 5 and 18 years (*n* = 20 experimental group), who were asked to practice GMI-based MI exercises, in addition to conventional therapy, for a total of 5 days/week for 4 weeks. The effectiveness of the intervention was assessed with the visual analog scale (VAS), a survey on catastrophizing and the accelerometer for physical activity and revealed a positive effect on pain symptoms only in a small number of patients.

The effect of MI was analyzed to treat SY in a case series (Biglioli et al., 2017). Authors proposed an intervention for patients aged between 12 and 47, lasting 3 weeks, to test post-facial surgery rehabilitation. Tools included internal trials of MI of specific movements. Treatment efficacy was tested by electromyography (EMG) and the Housee Brackmann scale (HB). The results revealed a significant improvement in facial movements in all patients (Biglioli et al., 2017).

MI based interventions in ST were evaluated in one original article (Lu et al., 2020). The study was conducted on a large mixed age-related population (patients from 16 to 70 years of age) to verify the efficacy of MI-based continuous passive movement control (CPM) and a brain-computer interface (BCI) in the recovery of a wrist extension following stroke. The duration of the intervention was 6 weeks. The efficacy was measured by calculating the range of motion by means of the EEG and by some indirect tests such as the Barthel index, measuring the degree of the assistance required. The intervention showed beneficial effects.

A completed clinical trial retrieved from online registers developed a BCI-based robotic arm and self-guided neurorehabilitation protocol for patients with SCI aged 14 years and older (NCT02443558, 2015). Objective of the study was to allow patients to interact with the robotic arm by modulating their own brain waves through kinesthetic MI (kMI) and visual MI (vMI) practices, using a portable EEG device (Emotiv EPOC). Published results refer exclusively to adult subjects, while participants appeared to perform better using vMI rather than kMI as an imagery modality for BCI control, the analysis did not prove a statistically significant correlation (Athanasiou et al., 2017). A rehabilitation system (iCTuS-L, Interactive Computer-based Therapy System for legs) was the goal of another completed clinical trial (NCT02149186, 2014) where AO, MI and EM, based on gaming sessions in virtual reality (VR), were used to treat NP and motor dysfunction in patients aged between 16 and 80 years with an incomplete SCI or stroke. The published results report data only from the adult population revealing beneficial functional training effects in subjects with chronic SCI (Villiger et al., 2017).

For sports-related brain concussions (SRBC), we recovered only one original work proposing a rehabilitative treatment for adolescents. The protocol included physical exercises, mental simulation of motor actions and positive imagination (Gagnon et al., 2016). Intervention efficacy was monitored using the Post-Concussion Scale (PCS), Beck Depression Inventory-2 (BDI-2), Pediatric Quality of Life Multidimensional Fatigue Scale (PQLMFS), Bruininks-Oseretsky Test of Motor Proficiency-2 (BOT), ImPACT^®^ for Cognitive Functioning, and the State Trait Anxiety Inventory (STAI). Postoperatively, the authors report that post-concussive symptoms, fatigue and mood improved.

Lastly, more recent anecdotal/single clinical studies retrieved from online registers and currently open, focus on congenital or acquired conditions with nervous system impairment opening the way to additional possibilities of therapies for complex disorders such as cerebellar ataxia (CA) secondary to resection of medulloblastoma (NCT04790981, 2021) and Duchenne Muscular Dystrophy (NCT05601986, 2022).

To summarize, this systematic review evaluates the use of MIT interventions in a heterogeneous group of children and adolescents with common and rare chronic disorders, recognizing genetic or acquired causes, presenting with various level of impairment in motor performance, planning and control, perceptual/sensory, behavior and executive functions.

Due to this array of conditions, severity, course and dysfunctions including the way of assessment of MIT, pooling the data was not achievable as intended. However, consistent with the objective of this review some common characteristics and comments can be outlined.

In practice, our results suggest that MI training is a potential resourceful approach to neurorehabilitation that enhances motor skills and coordination, making it particularly beneficial for conditions like DCDs and CP, which are among the most common neurodevelopment disorders. MI also bolsters cognitive functions, improving memory, attention, planning and problem-solving abilities, which is especially valuable for children with neurodevelopmental conditions. If integrates into tailored traditional rehabilitation programs, combining physical practice with mental rehearsal, MI interventions could expedite recovery and maximize the quality of life of young persons. Utilizing adaptive technologies such as VR and BCI systems provides real-time feedback, making rehabilitation exercises engaging and motivating. MI’s individualized approach recognizes each patient’s uniqueness, allowing therapy customization based on age, condition, and cognitive abilities. The potential outcomes of MI training on symptoms in comorbidity, including social/emotional manifestations, must be considered an extra value in pediatric neurorehabilitation. Moreover, MI facilitates progress traceable through assessment tools, ensuring that rehabilitation programs can be adjusted as needed for optimal outcomes. According to current evidence, introducing MI training at an early age offers short-term benefits, particularly for children with neurodevelopmental disorders, as it enhances motor learning and cognitive development. Additionally, MI shows promise in managing chronic pain conditions by empowering patients to reduce pain perception and improve overall well-being.

At the same time, some *limitations* have been recorded: to date, relatively few studies have explored the usefulness of MI interventions in the pediatric age; in many interventions distinguished on the base of their construct, there was not clear difference between the use of the terms “motor imagery” and “mental imagery,” reinforcing the consideration that these terms are often confused in the literature as in the clinical practice.

Moreover, disappointingly, beside the frequent inconsistent size of the population studied, the scarcity of RCTs consisting of clinical studies with in hand results, the great heterogeneity of protocol of interventions including the presence and/or suitability methods/scales adopted to evaluate the efficacy, close correlation with the severity of condition, reliability of MI mainly as add-on therapy and poor assessment of duration of efficacy, are restrictions that make the evidence gathered rather unpredictable, thus hampering the identification of decisions and evidence-based criteria for intervention planning and clinical practice recommendation leaving some unanswered questions that we were unable to address. An additional intrinsic limit of MI is that the therapist does not exactly know what the child is imagining. At this end, BCI or dynamic versions of MI (in which imagery is coupled with simplified patterns of the movements imitating some temporal or spatial features of the simultaneous mental representation of the action, such as stepping in place during walking imagery) have been proposed to compensate for this limitation.

## Conclusion

The systematic literature review results hereby presented revealed that MI training, integrated into neurorehabilitation programs, thus far shows an encouraging trend of positive outcomes in term of sensory-motor, mental, and social well-being, in children and adolescents with particular conditions/disorders of the nervous system. To gain larger and more satisfactory results, MI protocols should provide as much treatment as possible in terms of frequency, duration, and intensity of the most appropriate form of MI training required by the clinical condition.

Neurosciences supply fascinating evidence revealing how newer interventions could contribute to change the brain structurally and functionally; however, with no complete understanding of how an intervention acts, as in the case of MI, to ascribe the causal relationship still remains difficult.

In this respect, the possibility to evaluate the quality of MI in different neurodevelopmental disorders could uncover more neurobiological insights explaining why effectiveness of MI differs for each of them. It might be obvious to think that the greater the involvement of the motor areas, the greater the effectiveness of MI intervention. However, as discussed in this article, such an explanation would be considered rough. Certainly, the reason for such differing effectiveness should be sought in the complexity of MI networks and pathways which interplay with the heterogeneous pathogenic mechanisms underlying some of the neurodevelopmental disorders for which MI interventions are effective (e.g., cerebral palsy spectrum disorder, autism spectrum disorders) (Ruggieri, 2024).

MI assessment offers interesting opportunities for modeling feasible therapeutic interventions to promote motor learning or re-learning, ameliorate psychomotor skills and enhance cognitive performances, as proven in a consistent group of children with neurological disorders. These results provide also additional evidence on the assumption that exercises based on MI might be also combined to physical education and sport activities in non-therapeutic settings letting a quantitative and qualitative extension of interventions in the everyday life of disabled children.

The clinical use of MI for pediatric neurorehabilitive purposes, is however highly dependent on the complexity of MI mechanisms, which are specifically related to the underlying neurodevelopmental disorder. Thus, the precise neuropathophysiology of the treated child must be contemplated for tailored MI-based neurorehabilitation programs.

## Author contributions

AEG and SR searched the literature, analyzed the results supported by ED and drafted the first version of manuscript. MR, MI, CMV, VP, AEG, AP, and SR developed the theoretical framework. MR re-drafted the further versions of manuscript. AP contributed to the design and implementation of the research, re-drafted, and revised all the versions of manuscript. All authors contributed to the article and approved the submitted version.
