# Supplementary material for: Motor imagery for paediatric neurorehabilitation: how much do we know? Perspectives from a systematic review
Source: Front Hum Neurosci. 2024 Mar 20;18:1245707. doi: 10.3389/fnhum.2024.1245707 (PMC10987782; doi:10.3389/fnhum.2024.1245707)
Supplement: Supplementary file 1 [file Table_1.pdf]

NEURODEVELOPMENTAL CONDITIONS	REFERENCES	STUDY DESIGN	LEVEL OF EVIDENCE	PEDro SCORE	PARTICIPANTS			MI INTERVENTION			MEASUREMENT TOOLS (Pre- and/or Post-intervention)	OUTCOME		EFFICACY
					Sample size/no. (sex)	Age range (y.o.)	Control group	Treatment duration/sessions	Tasks	Type		Evaluation time	Assessment§	
**ADHD**	NCT05208255, 2022	RCT	2	/	- n/a (all)	7-12	Y	- 6 weeks<br>- 2 sessions/week	Imagined-NEP tasks; Timed Up and Go-Mental Chronometer	*Conventional*	Movement Imagery Questionnaire-Revised Second Edition; Kinesthetic and Visual Imagery Questionnaire; Timed Up and Go-Mental Chronometer; Ruler Drop Test; Conners Parent Rating Scale-Revised Short; dynamic balance and functionality evaluation; Stroop test; attention tasks	- Pre-post	(see *Measurements column)*	n/a
**ASD**	IRCT201812110 41929N1, 2019	RCT	2	/	- 30 (n/a)	8-13	Y	- n/a weeks<br>- 5 sessions	Motor imagery training (n/a)	*Conventional*	Movement assessment battery for children	- Pre-post	(see *Measurements column)*	n/a
**CA**	NCT04790981, 2021	RCT	2	/	- 50 (n/a)	7-9	Y	- 12 weeks<br>- 36 sessions	Modelling (mental simulation of an observed action)	*Ad hoc*	Assessment of severity of ataxia, and motor function parameters	- Pre-post	(see *Measurements column)*	n/a
**CP**	NCT02391324, 2015	RCT	2	/	- 160 (all)	5-18	Y	- 8-10 weeks<br>- 16-20 sessions	Motor imagery (n/a)	*n/a*	Gross Motor Function Measure (GMFM-66); Assessment of several clinical aspects and changes in physical/motor parameters (see *ref.)*	- Pre-post<br>- At follow-up after 3 months	(see *Measurements column)*	n/a
	NCT02754128, 2019	RCT	2	/	- 20 (all)	7-17	Y	- 6 weeks<br>- 16 sessions	Motor imagery (n/a)	*n/a*	Mental chronometry; Motor Imagery Questionnaire for Children; Pictorial Children's Effort Rating Table (PCERT); Assessment of changes in several physical/motor parameters (see *ref.*); fMRI; DTI	- Pre-post<br>- At follow-up after 4 weeks	(see *Measurements column)*	n/a
	NCT02584491, 2019	Before-and-after study (Non-rand.; single assign.)	4	/	- 9 (all)	7-17	/	- 6 weeks<br>- 16 sessions	Motor imagery (n/a)	*n/a*	Mental chronometry; Assessment of changes in several physical/motor parameters (see *ref.*); fMRI; DTI	- Pre-post<br>- At follow-up after 4 weeks	(see *Measurements column)*	n/a
	NCT03954808, 2020	RCT	2	/	- 51 (all)	7-18	Y	- 8 weeks<br>- n/a sessions	Motor imagery (n/a)	*n/a*	Movement Imagery Questionnaire-Children; Mental Chronometry for Timed Up and Go Test; Mental Chronometry for 10-Meter Walk Test; Mental Chronometry for Five Times Sit to Stand Test; Motor imagery entertainment scale; Motor imagery clarity scale; Assessment of changes in several physical/motor parameters (see *ref.*); mini-mental for children; EMG	- Pre-post<br>- At follow-up after 6 weeks	(see *Measurements column)*	n/a
	NCT04765917, 2021	RCT	2	/	- 50 (all)	7-10	Y	- 3 months<br>- 36 sessions	Modelling (mental simulation of observed action)	*Ad hoc*	Kinematic analysis (Kinovea software via 2D gait analysis)	- Pre-post	(see *Measurements column)*	n/a
**CSI**	NCT02443558, 2015	Quasi-experimental (Non-rand.; parallel assign.)	3	/	- 20 (all)	>14	Y	- n/a	Kinesthetic Motor Imagery; Visual Motor Imagery (MI-BCI: Emotiv EPOC wireless; EEG software)	*Ad hoc (Digital)*	Greek translation of the Spinal Cord Independence Measure, version III (g-SCIM-III)	- Pre-post<br>- At follow-up after 1, 6 and 12 months	(see *Measurements column)*	n/a

**DCD**	NTR5471, 2015	RCT	2	/	- n/a (all)	7-12	Y	- 9 sessions of 45 min + weekly 4 x 10 min homeworks	Mental simulation of on-demand actions	*Ad hoc*	mABC-2; Motor Coordination Questionnaire; Video- analysis of the trained motor skills; hand rotation Task; the radial visual guided pointing task; Action planning task (sword task); Rapid online control	- Pre-post	(see *Measurements column)*	n/a
	NCT04176159, 2021	RCT	2	/	- 99 (all)	6-12	Y	- n/a weeks<br>- 20 sessions	Motor imagery	*n/a*	MABC; scales	- Pre-post	(see *Measurements column)*	n/a
**DMD**	NCT05601986, 2022	RCT	2	/	- n/a (M)	5-12	Y	- 8 weeks<br>- 2 days/week	MI + CT (Motor Imagery training: n/a)	*Convent ional*	Kinovea Gait Analysis, Timed Up and Go Test, 2 Minute Walking Test, Motor Function Rating Scale for Neuromuscular Diseases, timed performance tests, Pediatric Berg Balance Scale, Pediatric Fear of Fall Questionnaire (Ped-FOF)	- Pre-post	(see *Measurements column)*	n/a
**SCI**	NCT02149186, 2014*	Before-and-after study (Non-rand.; single assign.)	4	/	- 79 (all)	16-80	/	- 16 weeks<br>- n/a sessions	Motor imagery (iCTuS-L: Interactive Computer-based Therapy System for legs)	*n/a (Digital)*	Sensorimotor Assessment; TMS; transcutaneous electrical stimulation	- Pre-post	(see *Measurements column)*	n/a
**ST**	NCT00355836, 2006	RCT	2	/	- 135 (all)	n/a	Y	- 4 weeks<br>- n/a sessions	Mental simulation of affected limb action	*Ad hoc*	Action Research Arm Test (ARAT); Grip strength;<br>Nine hole pegboard task; Function Limitation Profile; Barthel Index; Recovery Locus of Control	- Pre-post	(see *Measurements column)*	n/a
	NCT02149186, 2014*	Before-and-after study (Non-rand.; single assign.)	4	/	- 79 (all)	16-80	/	- 16 weeks<br>- n/a sessions	Motor imagery (iCTuS-L: Interactive Computer-based Therapy System for legs)	*n/a (Digital)*	Sensorimotor Assessment; TMS; transcutaneous electrical stimulation	- Pre-post	(see *Measurements column)*	n/a

S1. ONGOING RCTs RETRIEVED FROM ONLINE REGISTERS (in alphabetical order): **ADHD**: Attention Deficit Hyperactivity Disorder; **ASD**: Autism Spectrum Disorders; **CA**: Cerebellar Ataxia; **CP**: Cerebral Palsy; **CSI**: Cervical Spine Injury; **CT:** Conventional Therapy; **DCD**: Developmental Coordination Disorder; **DMD**: Duchenne Muscular Dystrophy; **SCI**: Spinal Cord Injury; **ST**: Stroke; **TD**: Typical Development.

n/a: not available (not described data in the article); *****: the article refers to both SCI and ST conditions; **§**: In cases of overlap, please refer to the "Measurement Tool" column.
